# Renal osteodystrophy in Brazil: the skeleton calls for greater attention!

**DOI:** 10.1590/2175-8239-JBN-2025-E007en

**Published:** 2025-06-06

**Authors:** Carolina Aguiar Moreira, Sérgio Gardano Elias Bucharles

**Affiliations:** 1 Fundação Pró-Renal, Instituto Pró-Renal, Centro de Pesquisa Acadêmica, Laboratório de Histomorfometria Óssea, Curitiba, PR, Brazil.; 2 Universidade Federal do Paraná, Departamento de Clínica Médica, Curitiba, PR, Brazil.

It is estimated that 10% of the global population has chronic kidney disease^1^ (CKD), and that among these individuals, a substantial proportion - particularly those in more advanced stages - will develop some form of one of the most common complications of this disease: CKD-mineral and bone disorder (CKDMBD)^2^.

Until a few decades ago, the term CKD-MBD was broadly defined as renal osteodystrophy (ROD); however, since 2006, it has come to refer more specifically to abnormalities in bone volume, mineralization, and turnover observed in CKD patients^3^. Understanding CKD-MBD and the magnitude of its impact is a routine task for nephrologists, given its importance in generating high rates of fragility fractures, worsening cardiovascular disease, and increased overall mortality in CKD, factors that are undoubtedly exacerbated by this true syndrome^4,5^.

Renal osteodystrophy (ROD) is a well-known bone disease that affects a substantial number of patients undergoing renal replacement therapy. With the use of bone biopsy and histomorphometric analysis of bone tissue, ROD may be classified into high-turnover bone disease (osteitis fibrosa), mixed bone disease (remodeling disorder associated with mineralization defect), and low-turnover (or adynamic) bone disease. In addition, these analyses provide nephrologists with information on bone volume and possible metal accumulation (aluminum and/or iron toxicity)^6,7^. It’s all very clear and simple, isn’t it? Not quite.

In our country - eternally emerging and hopeful regarding the future - as in many other parts of the world, there are very few centers of excellence in the study of osteometabolism that perform this procedure and the histological assessment of bone biopsies. Consequently, the knowledge accumulated within this veritable minefield still raises many unanswered questions. Nevertheless, the efforts of a select group of researchers in the field have contributed to a greater understanding of ROD in Brazil, with the establishment of a noteworthy national bone biopsy database. Compiled and thoroughly analyzed in the renowned REBRABO, Carbonara et al.^8^ published in 2023 a comprehensive analysis (N = 386, 82% of patients on hemodialysis) of ROD in Brazil, revealing a high prevalence of osteitis fibrosa (42%), approximately 25% of patients presenting with low bone turnover disease, and a worrying and high rate of aluminum intoxication, identified in 36% of biopsied patients^8^. In addition, special attention was given to the high frequency of histomorphometric osteoporosis (bone volume <17%), observed in 54% of biopsies.

In the current edition of the Brazilian Journal of Nephrology, Coutinho et al.^9^ revisited this topic, which is so relevant to our specialty, by evaluating 250 bone biopsies performed in the state of Pernambuco over an extended observation period (2004–2021), specifically including patients with G5D CKD. Throughout this period, it is important to highlight that care for CKD-MBD has improved, with the advent of new pharmacological therapies provided by the public health system, greater understanding of how mineral metabolism variables impact bone health in the CKD population, and improvements in the quality of dialysis therapy.

In this publication, Coutinho et al.^9^ evaluated a population composed mainly of women (57%), relatively young (median age of 48 years old), on renal replacement therapy for 9 years (on average), and with a low prevalence of diabetes mellitus (7.6%). These epidemiological data differ somewhat from those observed in our most recent dialysis therapy surveys, published by the Brazilian Society of Nephrology, in which men in older age groups are predominant, in addition to hypertensive and diabetic patients^10^.

Additionally, these patients had a high prevalence of fragility fractures, a history of aluminum-based phosphate binders (a therapy frequently used for decades but no longer recommended), and median parathyroid hormone (PTH) values > 1.000 pg/ml, which corroborated relevant information in the study, such as the high prevalence of osteitis fibrosa (54%) and aluminum toxicity (46%), in agreement with the previous analysis of REBRABO^8^. The prevalence of aluminum deposition remains high, raising concerns due to the multiple potential sources of contamination with which our patients may come into contact. Similarly, high mean PTH values are also observed in national surveys conducted annually by the BSN, with 18% of patients presenting PTH values > 600 pg/ml^10^.

The global picture in recent years has revealed a shift in ROD patterns, with the high prevalence of osteitis fibrosa being progressively replaced by low-turnover disease, likely due to epidemiological changes in the population currently developing CKD, primarily composed of older, diabetic, and inflamed patients^11^. However, the study by Coutinho et al.^9^ demonstrates that high-turnover disease, in which bone histomorphometry translates into increased resorption and cortical bone porosity, consequently leading to bone loss and microarchitectural deterioration, is still very common in our setting. As a clinical consequence, this leads to a high prevalence of fragility fractures, which exponentially increases morbidity and mortality in these patients and reduces their quality of life.

Although there are few national centers responsible for this kind of assessment (ROD) and a limited number of outpatient clinics specializing in the study of CKD-MBD in Brazil, we should encourage these professionals to continue providing us with valuable information obtained through this research, which decisively contributes to a better understanding not only of the variables of mineral metabolism, but also of dialysis therapy in the country. The objective is the prevention and early identification of adverse clinical scenarios that determine overall morbidity and mortality, such as fragility fractures and cardiovascular disease, highly prevalent in this very special patient population.

The present study demonstrates that, even in the era of artificial intelligence, bone histomorphometry remains useful in the diagnosis of ROD and essential in research aimed at a greater understanding of bone metabolism impairment secondary to renal failure. In addition, based on the biopsy results reported in the study, it is concluded that bone involvement in CKD still requires attention and care.
