# Supplementary material for: Renal osteodystrophy in Brazil: the skeleton calls for greater attention!
Source: J Bras Nefrol. 2025 Jun 6;47(2):e2025E007. doi: 10.1590/2175-8239-JBN-2025-E007en (PMC12143807; doi:10.1590/2175-8239-JBN-2025-E007en)
Supplement: Translation

# Translation: Osteodistrofia renal no Brasil: o esqueleto clama por mais atenção!

**DOI:** 10.1590/2175-8239-JBN-2025-E007pt

**Authors:** Carolina Aguiar Moreira, Sérgio Gardano Elias Bucharles

**Affiliations:** 1 Fundação Pró-Renal, Instituto Pró-Renal, Centro de Pesquisa Acadêmica, Laboratório de Histomorfometria Óssea, Curitiba, PR, Brasil.; 2 Universidade Federal do Paraná, Departamento de Clínica Médica, Curitiba, PR, Brasil.

Estima-se que 10% da população mundial apresente doença renal crônica^1^ (DRC) e que, entre esses indivíduos, uma parcela substancial - particularmente aqueles em seus estágios mais avançados - desenvolva alguma forma de uma das complicações mais frequentes dessa doença: o distúrbio mineral e ósseo associado à DRC (DMO-DRC)^2^.

Até algumas décadas atrás, a terminologia DMO-DRC era mais amplamente conceituada como osteo­distrofia renal (ODR), passando esta, a partir de 2006, a referir-se mais especificamente às alterações de volume, mineralização e remodelação ósseas observadas em pacientes com DRC^3^. Compreender o DMO-DRC e a magnitude de seu impacto é tarefa rotineira do nefrologista, em razão de sua importância na geração de elevadas taxas de fraturas por fragilidade, agravamento de doença cardiovascular e aumento da mortalidade geral na DRC, fatores indiscutivelmente potencializados por essa verdadeira síndrome^4,5^.

A osteodistrofia renal (ODR) é uma doença óssea bem conhecida, que acomete um número substancial de pacientes em terapia renal substitutiva. Com o auxílio de biópsia óssea e análise histomorfométrica do tecido ósseo, a ODR pode ser classificada em doença óssea de alta remodelação (osteíte fibrosa), doença óssea mista (alteração de remodelação associada a defeito de mineralização) e doença óssea de baixa remodelação (ou adinâmica). Além disso, essas análises municiam o nefrologista com informações a respeito do volume ósseo e eventuais impregnações por metais (intoxicação alumínica e/ou por ferro)^6,7^. Tudo muito claro, muito simples, não é mesmo!? Nem tanto.

Em nosso país, eternamente emergente e esperançoso quanto ao futuro, assim como em muitas outras partes do mundo, há pouquíssimos centros de excelência no estudo do osteometabolismo que realizam esse procedimento e a avaliação histológica de biópsias ósseas. Consequentemente, o conhecimento acumulado dentro desse verdadeiro campo minado ainda apresenta muitas questões em aberto. Contudo, os esforços de um seleto grupo de pesquisadores na área têm contribuído para um maior entendimento da ODR no Brasil, com a criação de um interessante banco de dados de biópsias ósseas realizadas em território nacional. Compiladas e muito bem analisadas no nominado REBRABO, Carbonara et al.^8^ publicaram, em 2023, uma abrangente análise (N = 386, sendo 82% pacientes em hemodiálise) da ODR no Brasil, evidenciando elevada prevalência de osteíte fibrosa (42%), cerca de 25% dos pacientes apresentando doença óssea de baixa remodelação e uma preocupante e elevada taxa de intoxicação alumínica, identificada em 36% dos pacientes biopsiados^8^. Além disto, foi dada atenção especial à elevada frequência de osteoporose histo­morfométrica (volume ósseo abaixo de 17%), observada em 54% das biópsias.

Na edição atual do Jornal Brasileiro de Nefrologia, Coutinho et al.^9^ revisitaram esse tema tão importante para a nossa especialidade, por meio da avaliação de 250 biópsias ósseas realizadas no estado de Pernambuco, ao longo de um amplo período de observação (2004–2021), incluindo especificamente pacientes DRC G5D. Ao longo desse tempo é importante ressaltar que o cuidado com o DMO-DRC foi se aperfeiçoando, com o advento de novas terapias farmacológicas disponibilizadas pela rede pública de saúde, maior compreensão de como as variáveis do metabolismo mineral impactam a saúde óssea da população com DRC e melhorias na qualidade da terapia dialítica.

Nesta publicação, Coutinho et al.^9^ avaliaram uma população majoritariamente composta por mulheres (57%), relativamente jovem (com mediana de idade de 48 anos), em terapia renal substitutiva há 9 anos (em média) e com baixa prevalência de diabetes mellitus (7,6%), informações epidemiológicas algo distintas daquelas que observamos em nossos mais recentes censos de terapia dialítica, publicados pela Sociedade Brasileira de Nefrologia, nos quais predominam homens, em faixas etárias mais avançadas, além de pacientes hipertensos e diabéticos^10^.

Adicionalmente, esses pacientes apresentaram uma elevada prevalência de fraturas de fragilidade, histórico de uso de quelantes de fósforo à base de alumínio (terapia frequentemente utilizada há décadas, mas atualmente não mais recomendada) e valores medianos de paratormônio (PTH) > 1.000 pg/ml, o que corroborou informações relevantes do estudo, como a elevada prevalência de osteíte fibrosa (54%) e intoxicação por alumínio (46%), em concordância com a análise prévia do REBRABO^8^. A prevalência de impregnação alumínica permanece alta, gerando preocupação devido às múltiplas fontes potenciais de contaminação com as quais nossos pacientes podem ter contato. Paralelamente, valores médios elevados de PTH também são observados nos censos nacionais elaborados anualmente pela SBN, com 18% dos pacientes apresentando valores de PTH > 600 pg/ml^10^.

O panorama mundial nos últimos anos tem revelado uma mudança nos padrões de ODR, com a elevada prevalência de osteíte fibrosa sendo progressivamente substituída pela doença de baixa remodelação, provavelmente devido às mudanças epidemiológicas da população que atualmente desenvolve a DRC, composta principalmente por pacientes mais idosos, diabéticos e inflamados^11^. Entretanto, o estudo de Coutinho et al.^9^ demonstra que a doença de alta remodelação, na qual a histomorfometria óssea se traduz em aumento da reabsorção e porosidade do osso cortical que, consequentemente, levam à perda óssea e deterioração da microarquitetura, ainda é muito frequente em nosso meio. Como consequência clínica, isso leva a uma elevada prevalência de fraturas por fragilidade, o que aumenta exponencialmente a morbimortalidade desses pacientes e reduz sua qualidade de vida.

Embora existam poucos centros nacionais responsáveis por esse tipo de avaliação (ODR) e um número limitado de ambulatórios especializados no estudo do DMO-DRC no Brasil, devemos estimular esses profissionais a continuarem nos municiando com valiosas informações obtidas por meio dessas pesquisas, que decisivamente contribuem para um melhor entendimento não apenas das variáveis do metabolismo mineral, mas também do cuidado com a terapia dialítica no país. O objetivo é a prevenção e a identificação precoce de cenários clínicos adversos, determinantes de morbimortalidade geral, como as fraturas de fragilidade e a doença cardiovascular, altamente prevalentes nessa população tão especial de pacientes.

O presente estudo demonstra que, mesmo na era da inteligência artificial, a histomorfometria óssea permanece útil no diagnóstico da ODR e essencial nas pesquisas voltadas a um maior entendimento do comprometimento do metabolismo ósseo secundário à falência renal. Adicionalmente, com base nos resultados das biópsias reportadas no estudo, conclui-se que o comprometimento ósseo da DRC ainda demanda atenção e cuidado.
